# Draft Genome Sequence of Rhodopirellula baltica Strain BR-MGV, a Planctomycete Isolated from Brazilian Mangrove Soil

**DOI:** 10.1128/MRA.01102-18

**Published:** 2018-10-11

**Authors:** Juliana Eschholz de Araújo, Rodrigo Gouvêa Taketani, Michele de Cássia Pereira e Silva, Marcus Venicius de Mello Lourenço, Fernando Dini Andreote

**Affiliations:** a Soil Microbiology Laboratory, Luiz de Queiroz College of Agriculture, University of São Paulo–ESALQ/USP, Piracicaba, São Paulo, Brazil; Louisiana State University

## Abstract

Members of the phylum Planctomycetes, which are capable of surviving in a wide range of environments, are some of the least-explored bacteria. Here, we report the near-complete draft genome sequence and annotation of the planctomycete Rhodopirellula baltica BR-MGV, which was isolated from the soil of a contaminated Brazilian mangrove.

## ANNOUNCEMENT

Planctomycetes is one of the least-explored bacterial phyla, mainly due to the difficulty of growing it in pure culture. These bacteria are taxonomically allocated in a superphylum named PCV (Planctomycetes, Chlamydiae, Verrucomicrobia) (1). This superphylum is located along the evolutionary separation between the Eukarya and Bacteria domains (1–3). Recent studies about this phylum have described new classes, orders, and genera (4–6). Although members of the phylum Planctomycetes normally exhibit low abundance in the environment, they have been found in a wide variety of terrestrial and aquatic habitats, reflecting their adaptation to different lifestyles (5, 7).

The present work was conducted in order to cultivate planctomycetes from a polluted mangrove soil, collected in the city of Bertioga (State of São Paulo, Brazil) (8). These mangroves are polluted by oil spills and other anthropogenic contamination. This isolate was obtained by cultivation on M13 medium plates with vitamin B complex solution, metal solution (Metals 44), and salt solution (Hutner’s salt) (7). The plate was kept at 19°C for 50 days. Isolated colonies were grown in the same medium prior to DNA extraction using the Wizard genomic DNA purification kit (Promega).

The genome sequencing of R. baltica strain BR-MGV was performed using the Ion Torrent PGM platform (Thermo Fisher Scientific). Briefly, the extracted genomic DNA was enzymatically fragmented and ligated to the appropriate adapters using the Ion Xpress Plus fragment library kit following the manufacturer's protocol. The obtained fragments were then ligated to the ionic beads and amplified by emulsion PCR using the Ion PGM template OT2 400 kit. The enriched beads were then sequenced on the Ion Torrent (PGM) platform using 316 chips and an Ion PGM 400 sequencing kit. The 3,207,713 reads that were obtained were subjected to quality control with FastX version 0.0.13 within Galaxy (https://usegalaxy.org) using the default settings, yielding a total of 3,073,229 quality-filtered reads. High-quality data were *de novo* assembled twice using SPAdes version 3.6.1 with two different *k*-mer ranges (first assembly, 21, 33, 55, and 77; second assembly, 21, 33, 55, 77, 99, and 127). These assemblies were integrated using CISA version 1.3. The resulting genome assembly consists of 99 contigs, with a longest size of 717,461 bp and an *N*_50_ size of 157,461 bp. The estimated genome size is 7.4 Mb, with a G+C content of 55.3%. The genome completeness rate estimated by CheckM was 94.6%, and the contamination rate was 1.18%, allowing its classification as being near complete with low contamination. Genome annotation was performed with PATRIC version 3.3.15, which identified 6,644 coding sequences (CDS) and 49 predicted noncoding RNAs (45 tRNAs and 4 rRNAs). The genome annotation of R. baltica strain BR-MGV has shown genes associated with the aromatic compound metabolism that occurs in the pathways for catabolism of aromatic compounds (1 gene for salicylate ester degradation), as well as genes associated with the metabolism of central aromatic intermediates (3 genes for salicylate and gentisate catabolism and 2 genes for the central metacleavage pathway of aromatic compound degradation). These genes are possibly involved in the biodegradation of central intermediates of hydrocarbon degradation pathways (9), suggesting the participation of this bacterium in the removal of contaminants in the mangrove soil.

### Data availability.

The genome project reported here has been deposited at DDBJ/ENA/GenBank under the accession number PPHI00000000 and to the Sequence Read Archive under accession number SRR7819959. The version described in this paper is the first version, PPHI01000000.
